# Development of a pre-clinical experimental simulation model of the natural porcine knee with appropriate ligamentous constraints

**DOI:** 10.1371/journal.pone.0216872

**Published:** 2019-05-14

**Authors:** Aiqin Liu, Eileen Ingham, John Fisher, Louise M. Jennings

**Affiliations:** 1 Institute of Medical and Biological Engineering, School of Mechanical Engineering, Faculty of Engineering, University of Leeds, Leeds, United Kingdom; 2 Institute of Medical and Biological Engineering, School of Biomedical Sciences, Faculty of Biological Sciences, University of Leeds, Leeds, United Kingdom; University of Memphis, UNITED STATES

## Abstract

A robust and stratified pre-clinical natural knee model, which has the capability to more appropriately simulate the biomechanical environment *in vivo*, will deliver more efficient and reliable assessment of soft tissue interventions before clinical studies. In order to simulate the biomechanical function of the natural knee without the natural ligaments in place, there is a requirement to develop appropriate spring constraints for the natural knee model. Therefore, this study was to investigate the effect of spring constraints on the function and output of the natural porcine knee model, and determine the spring constraint which most closely replicated the function of the natural ligaments. Two linear compression springs with stiffnesses of 9 N/mm (spring-9) and 20 N/mm (spring-20) were set at different free lengths in the anterior-posterior (A/P) axis in a natural knee simulator. The kinematic (A/P displacement) and tribological properties (shear force) output of the simulator were compared at different spring settings. The most appropriate spring setting was determined by comparing the A/P displacement and shear force output at different spring settings with those of the all ligaments model. Spring-9 with a free length of 4 mm showed the minimal difference (-0.03±0.68 mm) in A/P displacement output and spring-20 with a free length of 5 mm showed the minimal difference (-0.10±0.73 mm) in A/P displacement output compared to the all ligament control. There was no statistical difference between the two minimal differences either in A/P displacement or in shear force (paired t-test, p = 0.58, and p = 0.68 respectively) when both spring settings matched most closely to the A/P kinematics of the intact knee. This indicated that both conditions were appropriate spring constraints settings in the A/P direction for the natural porcine knee model.

## Introduction

Over eight million people in the UK suffer from osteoarthritis [1], which costs the National Health Service over £5 billion per year. The knee is the most common site for osteoarthritis, affecting 4.71 million people in the UK and this number is expected to rise considerably in the future [1]. Total knee replacement (TKR) has been relatively successful in treating late stage knee osteoarthritis in elderly patients [2–5], however, concerns persist with TKR for younger patients due to high rates of periprosthetic joint infections and aseptic mechanical failures reported in younger patients [6]. A recent study has revealed a high revision rate of 35% in patients aged 50–55 years [7]. Therefore, there is increasing clinical need for effective earlier stage surgical interventions, such as cartilage repair therapies and meniscal repair interventions, which replace or regenerate damaged or diseased soft tissue structures in the knee and therefore delay or prevent the requirement for total knee replacement surgery for patients. An essential evaluation step in the product development of any new regenerative device is to undergo an *in vivo* animal evaluation prior to human clinical studies, and *in vitro* animal models represent an important step to such an evaluation. There are, however, no standard pre-clinical test methods to assess the functional performance of these early stage interventions that can represent the biomechanical environment *in vivo* and also consider variations across patient groups.

In a previous study, a pre-clinical experimental simulation model using the porcine knee joint was developed to investigate the tribological function and biomechanics of the natural porcine knee [8]. This *in vitro* simulation model was shown to have the potential for investigation of the effect of knee structural, biomechanical and kinematic changes, as well as different soft tissue repair therapies on the tribological function of natural porcine knee joints. The model used a spring constraint in the anterior-posterior direction to simulate soft tissue function and to control kinematics and motions such as rolling and sliding. For future applications of this pre-clinical natural porcine knee simulation model, for example, the assessment of the functional performance of soft tissue repair interventions in the knee, it may be desirable to replace the ligaments with artificial spring constrained to minimise the variability from the model. Therefore, the determination of more appropriate artificial spring constraints is required to simulate the function of the natural ligaments in this model.

Numerous *in vitro* studies of TKRs have applied spring constraints in artificial knee simulators in order to simulate the soft tissue/ligament functions in the natural human knee which constrain anterior-posterior and tibial rotation movement [9–12]. Physical linear compression springs have been commonly used in force controlled knee simulators to provide soft tissue restraint for the human knee. Van Houtem [12] studied the accuracy of the soft tissue restraint in a force controlled knee simulator and applied a gap of 2.5 mm in the anterior-posterior direction between the spring and the tibia to simulate the toe region of the ligament load-displacement curve identified by Fukubayashi et al. [13]. Their study suggested that using springs of intermediate stiffness between 7.24 N/mm to 33.8 N/mm would improve the accuracy of simulating human knee motion. The ISO standard [14] for wear testing of TKR using force control parameters recommends the use of springs with a stiffness of 9.3 N/mm on both the ACL side and PCL side, and with a ± 2.5 mm gap set at the neutral position in the simulator to simulate the laxity of the natural human knee ligament. This was based on the studies of Haider [15, 16].

The setting of spring constraints in a knee joint simulator, including the stiffness and gap distance, affects the kinematics of the TKR specimen and therefore influences the friction and wear results [11, 17]. Similarly, for the natural porcine knee joint simulation model, the setting of the spring constraints influences the kinematics and would also affect the tribological performance of cartilage and meniscal repair and replacement therapies in the knee. Therefore, it is important to improve the precision of the spring restraint in the natural knee simulator in order to more appropriately simulate the biomechanical environment *in vivo*. This is necessary to deliver a more efficient and reliable assessment of soft tissue therapies before clinical trial. Currently, however, there have been no studies that have investigated spring constraints in the natural knee simulation model. Therefore, the aims of this study were to investigate the effect of input parameters of the anterior-posterior spring constraints on the output of the natural porcine knee simulation model including knee kinematics and tribological properties. Different spring settings, including stiffness and free length, were applied to the previously developed natural knee simulator [8]. The kinematics output (anterior-posterior (A/P) displacement) of the natural porcine knee and tribological behaviour in terms of shear force output at different spring settings were compared with those of the natural porcine knee with all the ligaments intact (control). The spring settings which showed the minimal difference in A/P displacement and shear force output were considered as the appropriate spring conditions for the natural knee simulation model.

## Materials and methods

### Single station knee simulator

**Fig 1:**
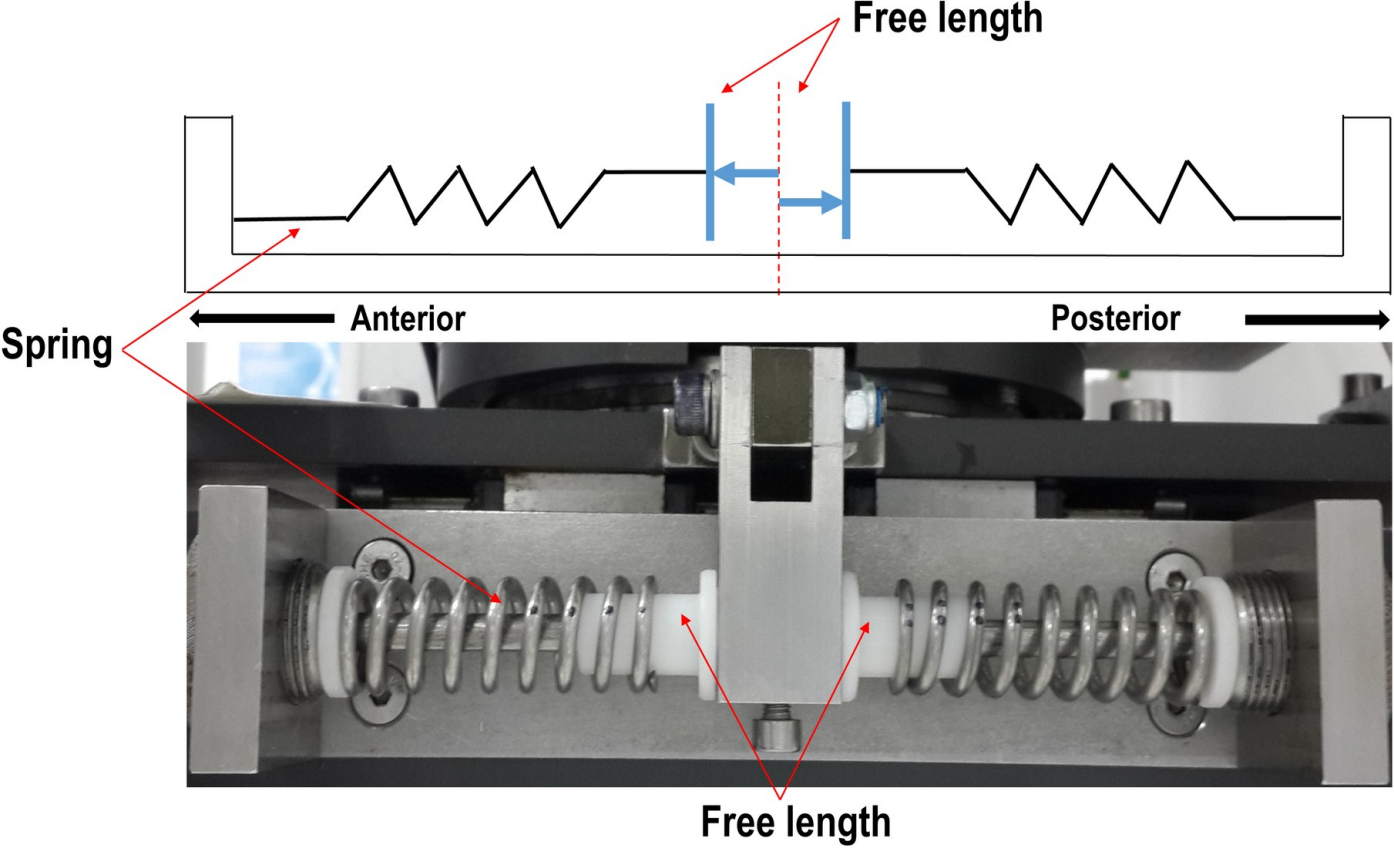
A/P spring configuration on the tibial side in the simulator.

This study used the single station natural knee joint simulator (Simulation Solutions, UK) which has been described thoroughly in our previous study [8]. The simulator is capable of recreating *in vivo* knee motions including sliding, rolling, and a combination of sliding and rolling motions, by controlling mechanical constraints in the simulator. However, in the previous study, the mechanical constraints were precompressed springs in the anterior-posterior side with no gap setting. Therefore, in order to more realistically simulate physiological soft tissues, a new spring configuration, which allowed the application of free length to the spring constraint, was designed and configured as shown in Fig 1. Two identical compression springs (Lee Spring Ltd, UK), as recommended by the ISO for situations in which both the PCL and ACL are sacrificed were inserted in both the anterior and posterior directions, which constrained the anterior and posterior movement of the natural knee joint.

### Porcine knee joint

Porcine knee joints of similar size taken from the right hind leg were obtained from 9 month old Large White pigs (John Penny & Sons, Leeds, UK) within 24 h of slaughter (n = 6). Each porcine leg was dissected by carefully removing soft tissues and leaving all ligaments, cartilage and meniscus in place. The centre of rotation was determined using a template methodology according to our previous study[18]. Both the femur and tibia were cemented in custom-build pots using polymethylmethacrylate (PMMA; WHW Plastics, UK) [8]. Throughout the procedure, the soft tissues were kept moist using phosphate buffered saline (PBS; MP Biomedicals, UK). The axial force axis was shifted medially by 0.07 of the tibial width (approximately 4.5 mm) by sliding the tibial pot in the fixture with the aim of causing greater medial compartment loading[14].

### Measurement of kinematic and A/P shear force in A/P direction

**Fig 2:**
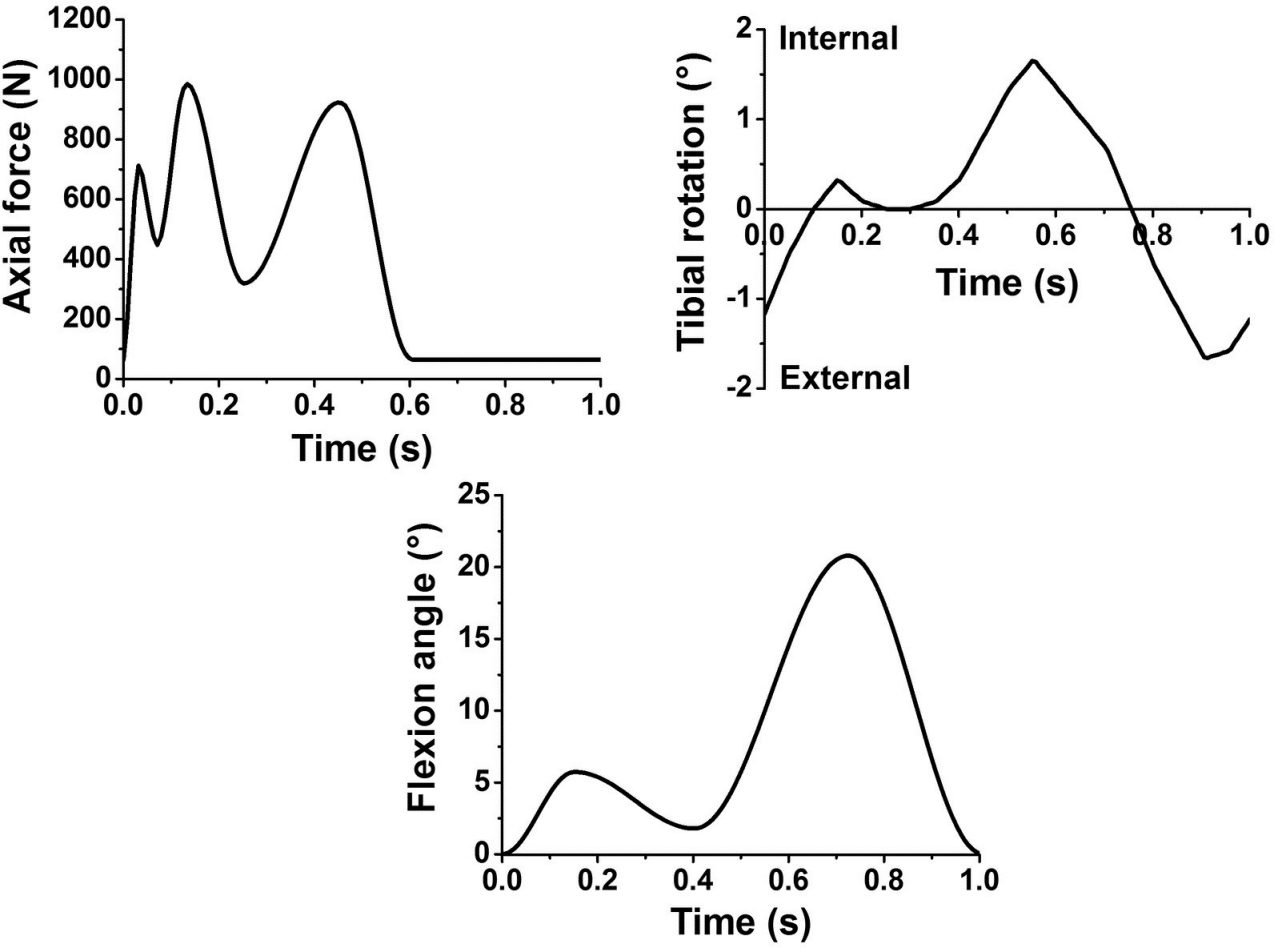
Porcine kinematic input profiles. The magnitude of the peak load in the load profile was 1000 N and the flexion angles varied from 0°to 21°. Tibial rotation was driven from -1.6° to 1.6°.

**Fig 3:**
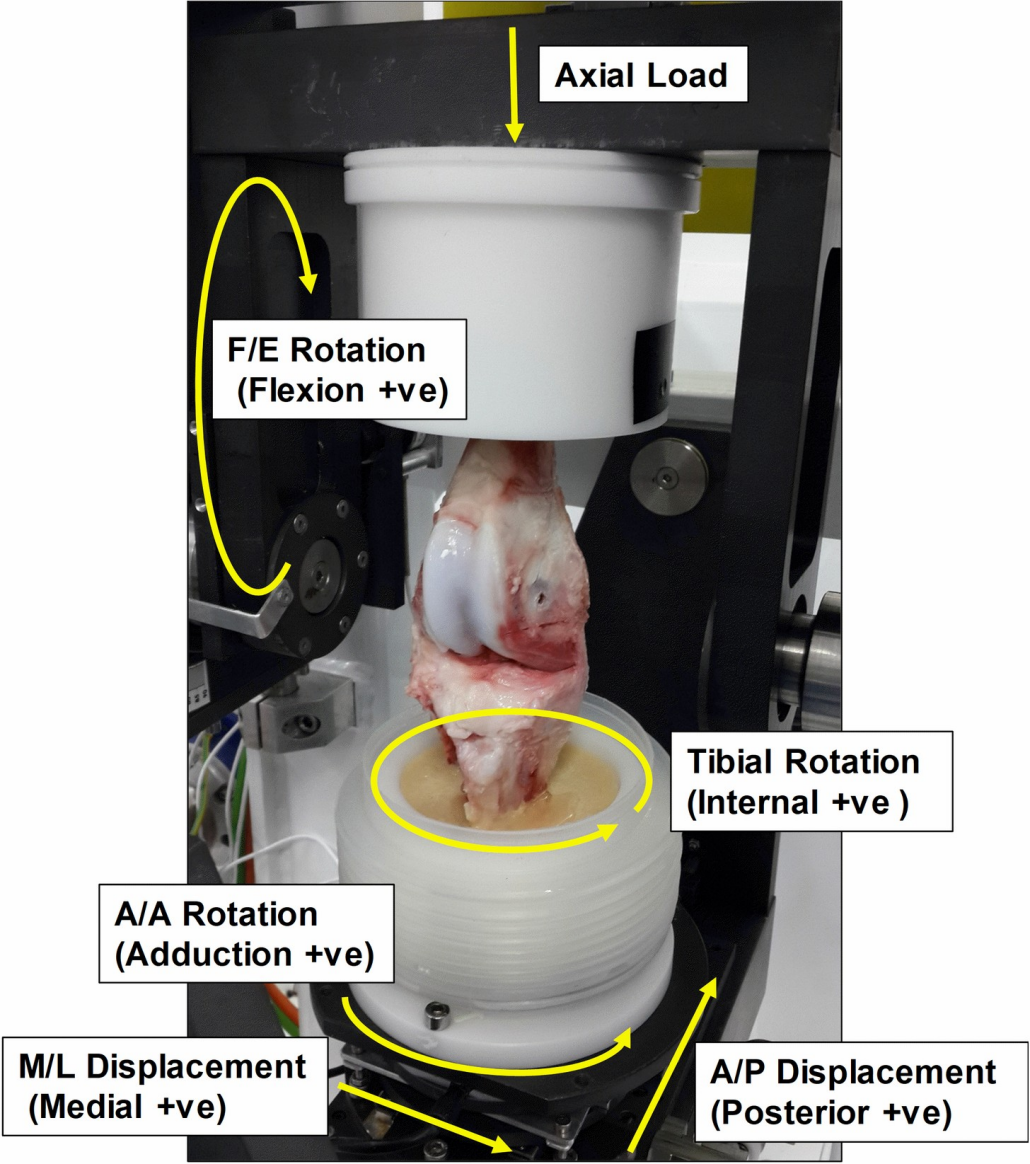
A schematic diagram of a tibiofemoral porcine knee joint mounted in the simulator with indications of the six degrees of freedom and polarities.

**Fig 4:**
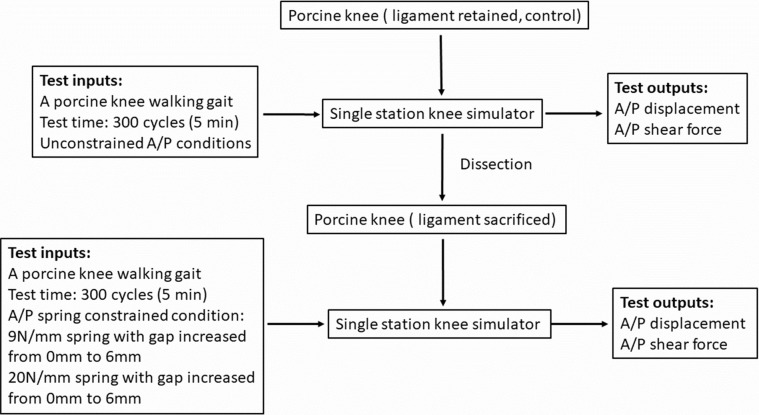
Flow chart of the test method for each porcine knee sample.

A porcine knee walking gait input profile [8] which was scaled from the high kinematic Leeds artificial knee input profiles to the kinematic limits of porcine knee tissue for axial load, internal/ external rotation and flexion/extension (all under displacement control), was applied in this study as shown in Fig 2. A schematic diagram of a tibiofemoral porcine knee joint mounted in the simulator with indications of the six degrees of freedom and polarities is shown in Fig 3. The abduction/adduction (A/A) motion was left unconstrained while the medial/lateral (M/L) displacement was constrained in all instances. The anterior/posterior (A/P) motion was not driven in any of the tests, however, the levels of A/P constraint were controlled by the spring configuration. The ultimate goal of the simulation model is to provide a platform to study the tribology of early human knee interventions, therefore, the spring constants applied in this study were according to references investigating human knee kinematics in knee simulators. The ISO standard [14] recommends the use of a spring with a stiffness of 9.3 N/mm for testing knee prostheses requiring resection of both cruciate ligaments. Therefore, a spring with a stiffness of 9 N/mm was chosen in this study. Furthermore, Van Houtem [12] studied the soft tissue restraint in a force controlled knee simulator and applied a gap of 2.5 mm in the anterior-posterior direction between the spring and the tibia to simulate the toe region of the ligament load-displacement curve. Their study suggested that using springs of intermediate stiffness between 7.24 N/mm to 33.8 N/mm would improve the accuracy of simulating human knee motion. Therefore, 20 N/mm as an intermediate stiffness between 7.24 N/mm to 33.8 N/mm was chosen in this study. Therefore, 9 N/mm and 20 N/mm, which represented these soft and hard springs were applied in this study in order to investigate the effect of the spring stiffness on the knee kinematics and tribological properties. A flow chart of the test method is shown in Fig 4. Initially, the kinematics in terms of A/P displacement and the tribological behaviour in terms of A/P shear force between the femur and tibia along the A/P axis, were determined for each porcine knee with all of the ligaments retained under fully unconstrained A/P conditions (no springs). Each test ran for 300 cycles at a frequency of 1 Hz, which allowed the tests to stabilise and for consistent data to be generated. All the ligaments were then sacrificed and a series of test conditions were applied to the spring configuration as shown in Table 1. Six samples were tested in total. In order to eliminate the bias of the test order, half of the samples were initially tested under spring-9 conditions with the gap increased from 0 mm to 6 mm and then spring-20 from the gap of 0 mm to 6 mm. The other half were tested initially under spring-20 and then spring-9 in the same order of gaps. The simulator outputs including A/P displacement and A/P shear force from each test were recorded and exported to an Excel spreadsheet for analysis which has been described in the previous study [8]. A/P displacement and A/P shear forces under each test condition (Table 1) at two different time points were compared to those under the all ligament retained conditions (all ligament control). The time points of 0.15 s and 0.72 s were chosen for the analysis for A/P displacement, as the A/P displacement reached its highest values resulting from the highest flexion input angles at these two time points.

**Table 1:** Description of the test conditions with respect to spring rates and free lengths of spring constraints.

Spring Rates	9N/mm (Spring-9)	20N/mm (Spring-20)
**Spring Free Lengths (±)**	0mm, 1mm, 2mm, 3mm, 4mm, 5mm, 6mm	0mm, 1mm, 2mm, 3mm, 4mm, 5mm, 6mm

A positive value (+) means spring free length set in posterior direction; A negative value (-) means spring free length set in anterior direction.

### Statistical analysis

The mean A/P displacement and A/P shear force with 95% confidence limits were calculated under the different test conditions and the all ligaments retained condition. The absolute values of A/P shear force and A/P displacement under the different test conditions were compared using one-way ANOVA. This was followed by the T-method [19] to determine the minimum significant difference (MSD; p< 0.05) between the control and test condition groups. The difference between each test condition group mean and the control group mean were compared to the MSD. A difference between the group means greater than the MSD value indicated a significant difference (p< 0.05).

The value of the difference (D) between each test condition and the control was calculated. The difference between D of each test condition was tested using the paired t-test. A significance level of p<0.05 was applied.

The dataset associated with this article is openly available from the University of Leeds Data Repository[20].

## Results

### Comparison of A/P displacement and A/P shear force output profiles

**Fig 5:**
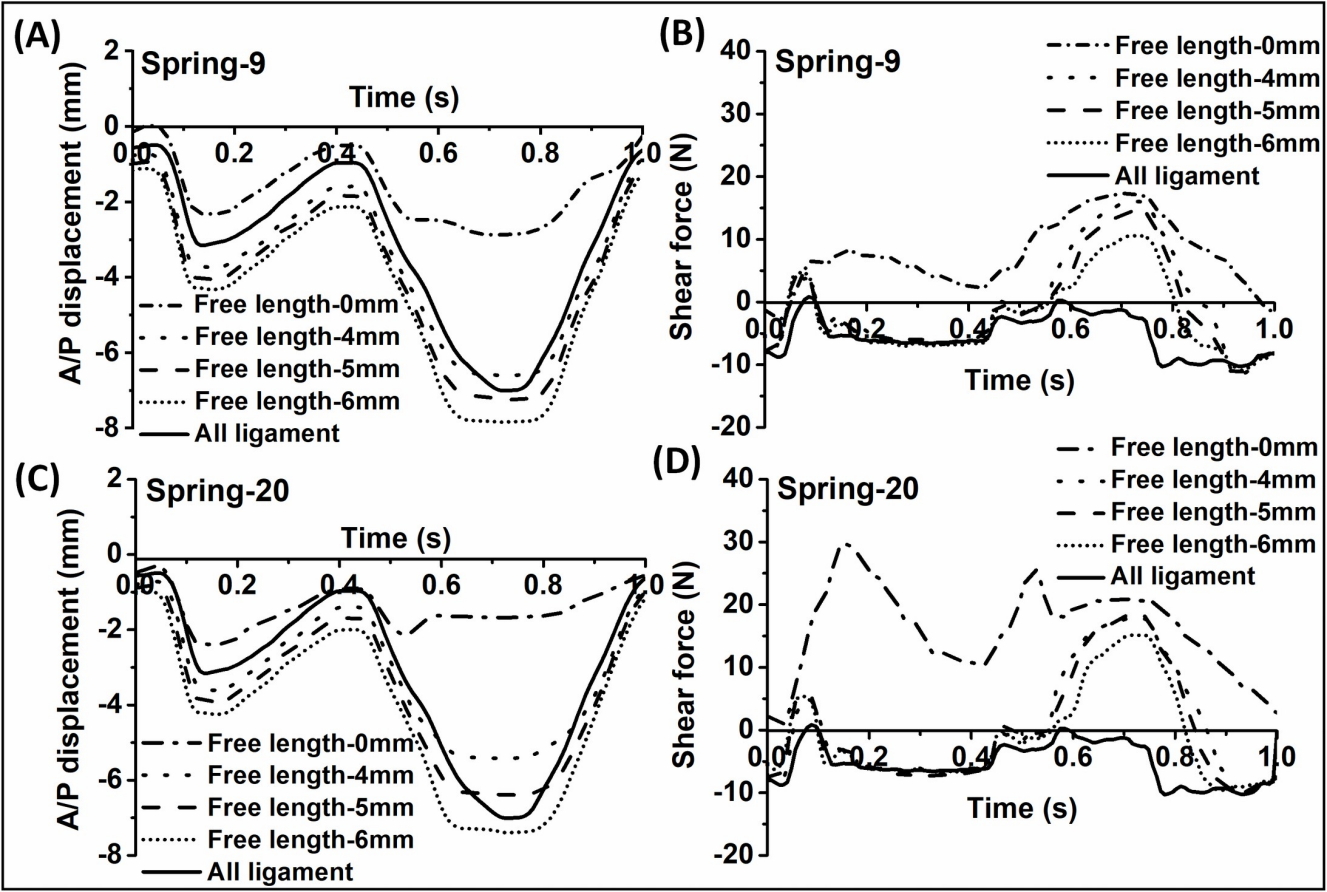
Typical kinematic output profiles of A/P displacement and A/P shear force from the intact knee (all ligaments retained) and different spring free length conditions. (A) and (B): spring-9; (C) and (D): spring-20. The anterior A/P displacement and anterior A/P shear force were taken to be negative. Only test conditions of 0 mm, 4 mm, 5 mm and 6 mm are shown. Data for other conditions are available through the University of Leeds Data Repository[20].

The A/P displacement profiles of both spring-9 and spring-20 showed similar shapes to the all ligament control at all spring conditions (Fig 5A and 5C) which followed closely the trend of the F/E input profile (Fig 2). As shown in Fig 5A and 5C, increasing the free length of both spring-9 and spring-20 caused an increase in the A/P displacement. For both springs, there were significant differences in the A/P displacement for different free lengths at the time point of 0.72 s (ANOVA, p<0.01). The peak value of the A/P displacement for spring-9 and spring-20 increased from 2.9 mm to 7.8 mm and 1.7 mm to 7.0 mm, respectively, when the free length increased from 0 to 6 mm. Spring-9 produced higher peak values of A/P displacement compared to spring-20 when both of the springs were set at the same free length. For spring-9, the maximum A/P displacements with the free length of 4 mm and 5 mm matched most closely to the A/P displacement of the all ligament control during the swing phase of the gait cycle (in the region 0.6–0.8 s). For spring-20, the maximum A/P displacements which were most closely matched to the A/P displacement of the all ligament control were at free lengths of 5 mm and 6 mm during the swing phase of the gait cycle. These results indicated that spring-20 with a higher spring stiffness required a larger free length setting compared to spring-9 in order to match the kinematic output of the natural knee.

On the other hand, the A/P shear force profile of spring-9 showed a similar trend to the natural knee at all settings, while the A/P shear force profile of spring-20 exhibited a similar shape to the natural knee only when free lengths were applied (Fig 5B and 5D). For both springs, increasing the free length caused a decrease in the shear force. There was a significant difference in the A/P shear force among different free lengths for spring-9 (ANOVA, p = 0.048) and spring-20 (ANOVA, p = 0.003) at the time point of 0.15 s. Spring-20 caused higher values of shear force and a different profile trend compared to the spring-9 when both of the springs were set at the free length-0mm condition, but the differences became smaller as the free length distance increased. Both of the springs generated high values of posterior shear force (positive value) at all of the free length settings while the all ligament intact knee produced a low value of anterior shear force, specifically during the swing phase of the gait cycle.

### Comparison of A/P displacement and A/P shear force at the two selected time points

**Fig 6:**
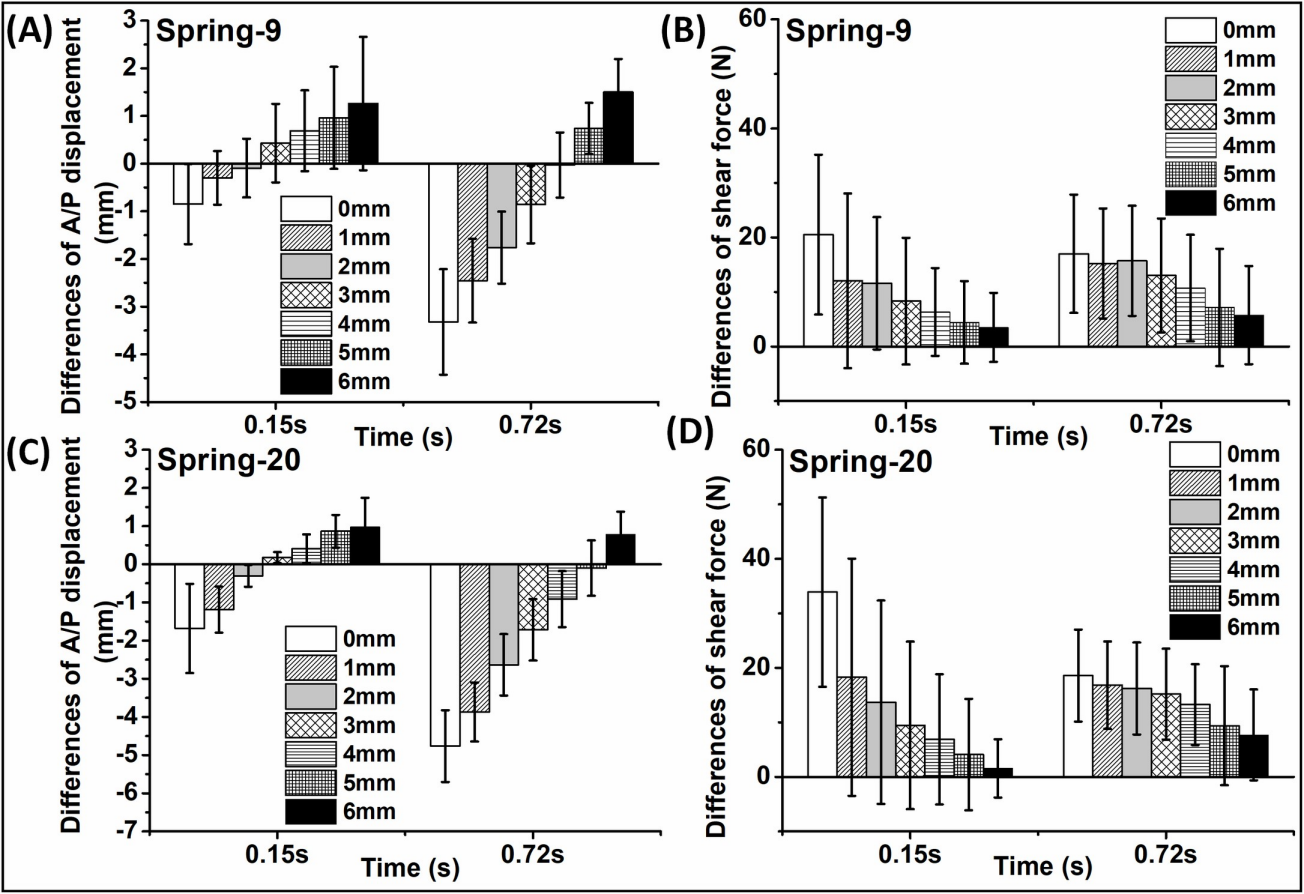
Comparison of differences of A/P displacement and A/P shear force under different free length conditions. (A) and (B): spring-9; (C) and (D): spring-20. Data is plotted as the mean of the differences between the test and the intact knee (n = 6) ± 95% confidence level. A negative value of the difference indicates that the value from the all ligament condition (intact knee) was higher than the value measured under each spring setting.

The differences in A/P displacement and A/P shear force from different spring conditions compared to those of the intact knee at the two selected time points is shown in Fig 6. For spring-9, a free length of 2 mm showed the minimal difference (-0.09±0.62 mm) in A/P displacement compared to the all ligament control at the time point of 0.15 s (Fig 6A). Spring-20 showed the minimal difference (0.17±0.14 mm) in A/P displacement when it was set at a free length of 3 mm compared to the all ligament control at the same time point of 0.15 s (Fig 6C). The data changed at different time points. For spring-9, a free length of 4 mm showed the minimal difference (-0.03±0.68 mm) in A/P displacement compared to the all ligament control at the time point of 0.72 s when the A/P displacement reached the peak value (-6.11±1.05 mm) during the gait cycle (Fig 6A). Spring-20 showed the minimal difference (-0.10±0.73 mm) in A/P displacement under the setting of 5 mm compared to the all ligament control at the time point of 0.72 s (Fig 6C). These findings were consistent with the results of the A/P displacement profile comparison which suggested that the larger free length was required for spring-20 with higher spring stiffness than spring-9 in order to match the A/P displacement output of the natural knee.

As shown in Fig 6B and 6D, increasing the free length of the spring resulted in a decrease in the differences in A/P shear force for both of the springs compared to the all ligament control at both time points. A free length of 6 mm showed the minimal difference of 4±6 N for spring-9 and 2±5 N for spring-20 in A/P shear force compared to the all ligament control at the time point of 0.15 s. Similarly, a free length of 6 mm demonstrated the minimal difference of 6±9 N for spring-9 and 8±8 N for spring-20 at the time point of 0.72 s. There were no significant differences (paired t-test, p = 0.50) in the shear force difference between spring-9 with a free length of 2 mm and spring-20 with a free length of 3 mm when both spring settings showed the minimal differences in the A/P displacement at the time point of 0.15 s. Similarly, there were no significant differences (paired t-test, p = 0.68) in the shear force difference between spring-9 with a free length of 4 mm and spring-20 with a free length of 5 mm when both spring settings showed the minimal differences in the A/P displacement at the time point of 0.72 s.

## Discussion

The aim of this study was to refine and optimise the existing natural porcine knee joint simulation model in terms of determining the most appropriate properties of the artificial ligamentous constraints. This was achieved through investigating the effect of spring constraints on the kinematic outputs and tribological properties of the natural knee in our simulation model. Fukubayashi et al [13] found that insufficient ligament constraints caused a significant anterior and posterior displacement compared to the natural knee. This change in kinematics would cause abnormal and non-physiological knee motion. Therefore, it was extremely important to determine appropriate spring constraints that would replicate the natural knee motion with intact ligaments. This will enable a more accurate assessment of the tribological performance of cartilage and meniscal repair and replacement knee therapies in the *in vitro* simulation system.

The all ligament model results showed that the porcine ligaments stabilised the knee while allowing some free A/P motion which caused minimal shear force measurement (<10 N) due to the laxity of the ligament structures. The A/P displacement profiles of all spring-9 and spring-20 test conditions followed similar trends to the all ligament control which closely matched the flexion angle input profile. These results indicated the appropriateness of using compression spring constraint, in order to simulate the kinematics of the natural porcine knee. Increasing the free length of the spring caused an increase in A/P displacement and a decrease in A/P shear force which indicated that a larger free length of the spring caused greater free motion of the knee in the anterior and posterior axis and contributed less to the A/P shear force measurement. These results indicated that the application of a combination of spring with free length was successful in simulating the natural porcine ligament function in this study. Different spring rates have been applied to force controlled knee simulators to simulate the soft tissue/ligament functions of human knees for *in vitro* studies of TKRs [10–12, 21]. Desjardins et al. [10, 21] used springs with a spring rate of 20 N/mm in the A/P direction for a force controlled knee simulator. Van Houtem suggested using springs of intermediate stiffness between 7.24 N/mm to 33.8 N/mm to improve the accuracy of simulating human knee motion. In order to understand the effect of the spring stiffness on the output of the natural porcine knee model, two different values of stiffness were compared which were selected according to the study by Van Houtem[12]. The results showed that both of the springs were able to closely match the A/P displacement profile of the intact knee when they were set at a certain free length. Spring-20, with higher spring stiffness, required a larger free length to match the A/P displacement profile of the intact knee compared to spring-9. The data also showed that spring-9, with a lower stiffness, generated smaller differences than spring-20 when both springs matched most closely to the kinematic of the intact knee, but the difference was not statistically different (paired t-test, p = 0.32 at 0.15s and p = 0.58 at 0.72s). Both of the springs achieved the minimal difference in A/P shear force compared to the intact knee when the springs were set at a free length of 6 mm. Spring-20 caused significantly higher value of differences in A/P shear force than spring-9 when no free length was applied (paired t-test, p = 0.03) at the time point of 0.15 s. However, the differences in the shear force between the two springs were not statistically significant under any free length spring conditions at any time points, which indicated that the effect of spring stiffness on the A/P shear force was not significant. On the other hand, there were significant differences in the A/P displacement among different free lengths for each spring at the time point of 0.72 s (ANOVA, p<0.01) and there was a significant difference in A/P shear force among different free lengths for each spring at the time point of 0.15 s (ANOVA, p = 0.048 for spring-9, p = 0.003 for spring-20) which indicated that the free length of the spring played an important role in both the kinematics and shear force output of the porcine knee. These results suggested that for the conditions studied the effect of the free length on the output of the natural porcine knee might be greater than the effect of the spring stiffness tested.

Spring-9 with a free length of 2 mm and spring-20 with a free length of 3 mm showed the minimal differences in A/P displacement compared to the all ligament control at the time point of 0.15 s. However, both settings gave significant differences in A/P displacement compared to the all ligament control at the time point of 0.72 s (T-Method). On the contrary, spring-9 with a free length of 4 mm and spring-20 with a free length of 5 mm showed small differences in A/P displacement compared to the all ligament control at the time point of 0.72 s. Both of these settings showed no significant differences in A/P displacement compared to the all ligament control at 0.15 s (T-Method). Therefore, spring-9 with a free length of 4 mm and spring-20 with a free length of 5 mm matched more closely to the kinematic profile of the all ligament condition. The results also showed no statistical difference between the differences in A/P displacement or shear force when both spring settings matched most closely to the kinematics of the intact knee at the time point of 0.72 s. It was concluded that spring-9 with a free length of 4 mm and spring-20 with a free length of 5 mm were both effective spring constraints settings to match the natural ligament constraints in the *in vitro* natural porcine knee model.

Both springs caused higher values of posterior shear force (positive value) at all of the free length settings compared to the intact knee which generated lower anterior shear force during the swing phase of the gait cycle. When all the ligaments were retained, the A/P shear force measured from the simulator was a combination of the reaction force between ligaments and the interaction force between the femur and tibia since the complex geometries move relative to each other, however, these forces were small in magnitude, which resulted in a small shear force. The situation was, however, changed when all the ligaments were removed and the spring constraints were introduced. During the swing phase of the gait cycle, all of the spring setting conditions generated larger anterior A/P displacement than its free length which indicated that the spring was compressed resulting in higher posterior shear force due to the resistance of the spring constraints. Interestingly, the response of the A/P shear force at different spring settings was different during 0.1 s to 0.6 s before the swing phase of the gait cycle. With increasing free length, the polarity of the measured shear force gradually changed from the posterior to the anterior direction which indicated that there was a transition from spring reaction force to interaction force of geometry in the porcine knee. These results indicated that changes of geometries to the femur and tibia which might be introduced by surgical or biomaterial intervention could be interpreted by the changes of measured shear force during this time phase.

The results showed that a larger free length caused lower shear force measurements and a higher stiffness of the spring required a larger free length to match closely to the kinematic profile of the natural knee which indicated that a higher stiffness spring might be a better option when investigating changes in the shear force induced by knee therapies. In this study, increasing the spring stiffness from 9 to 20 N/mm did not result in any significant differences in shear force measurements. Therefore, spring-9 and spring-20 were both good options for the natural porcine knee model. Applying a spring with stiffness higher than 20 N/mm, which will require a larger free length to match the kinematics of the all-ligament knee might result in a significant decrease in the shear force. However, applying a larger free length for a high stiffness spring will likely increase the free motion length of the natural knee without any physical constraints. This might adversely affect the stability of the knee and cause dislocation of the femoral and tibia joint. In this study, there was no separation of the articular surface observed when both of the springs were set at the largest free length of 6 mm. This was confirmed by the shear force results which did not show any spikes in the profile. Therefore, in addition to spring stiffness and free length, there are other factors such as the stability of the knee and limitation of the simulation such as the limit of physical A/P which also needed to be taken into account in selecting appropriate spring constraint conditions for the simulation of the natural knee joint.

This study has limitations. Firstly, the results presented in this study highlighted the influence of the spring constraints on the kinematic output and tribological properties of the porcine knee joint. The optimised spring conditions in the present study should not, however, be applied directly to the human knee model due to the different anatomy and kinematics of the human knee joint. Secondly, this study chose the sagittal plane to investigate primarily, as the primary function of the cruciate ligaments is in controlling the anterior-posterior motion. This study successfully developed the methodology to simulate ligament function in the sagittal plane in a porcine knee model, which provides a baseline for investigating ligament function further in a human knee model in the future. The future human knee model study will adopt the methodology from this study to investigate the mechanics further in the coronal and axial planes, which will provide a more comprehensive and reliable assessment of cartilage and meniscus repair and replacement interventions before *in vivo* studies.

This *in vitro* porcine model represents a key step on the product development pathway of new regenerative devices. This step provides valuable biomechanical and tribological functional performance details and is representative of the large animal model that the new regenerative device will subsequently be evaluated in in *in vivo* studies, prior to human clinical studies. These are essential evaluation steps for any new regenerative device. Further, the use of animal knee tissue is a necessary step in methodology development due to the high cost, ethical implications and scarcity of human knee specimens. Porcine knees were chosen because their joint size, joint loading, cartilage and trabecular bone thickness more closely match the human condition than alternative animal models [22]. Due to this anatomical similarity, the porcine knee model has previously been widely used in studying human knee joint disease such as ligament disorders [23, 24] and new treatments such as a novel meniscus repair technique [25]. Since our study was focused on methodology development, using a porcine knee model was an important step prior to investigating a human knee model. Since the source of the porcine knees was well controlled, with specimens originating from pigs in the same age range, this minimised biological variability and provided a consistent quality of tissue to work with. This compares with the wide range of age related changes and inconsistent tissue quality of human donor tissue. In the product development process, early inventions will always require pre-clinical testing in a large *in vivo* animal trial, and this is another reason why the use of an animal model is justified. In summary, the porcine knee model developed in this study does not only provide a baseline for developing a human knee model, but also is of great value in terms of representing an essential pre-human clinical animal model which is an essential evaluation step for any new regenerative device.

The findings from this study have provided important guidelines for selecting appropriate soft tissue constraints in the natural knee model with the aim of matching these constraints more closely to the constraints of the ligaments in individual joints. It also provides a baseline for developing a natural human knee model with appropriate spring constraints to simulate natural ligament function. Each individual natural human knee joint will have different knee kinematics as a result of variations in anatomy, soft tissue laxity and associated levels of disease such as osteoarthritis. Therefore, the appropriate constraints for the natural human knee joint will require investigation for each individual joint specimen to represent the biomechanical environment *in vivo*, and enable variations in that environment across the patient cohort to be simulated.

## Conclusions

A natural porcine knee simulation model with refined spring constraint conditions which more closely replicated the natural porcine ligament function was successfully developed in this study. The results indicated that many factors should be taken into account when selecting appropriate spring conditions including spring stiffness, free length, stability of the knee and limitations of the simulation. The development of a natural human knee model with refined spring constraint conditions is currently under investigation with the aim of assessing the effect of cartilage and meniscal repair and replacement therapies on the tribological function of the natural knee.
